# Dosing Recommendations for Vancomycin in Children and Adolescents with Varying Levels of Obesity and Renal Dysfunction: a Population Pharmacokinetic Study in 1892 Children Aged 1–18 Years

**DOI:** 10.1208/s12248-021-00577-x

**Published:** 2021-04-11

**Authors:** Cornelis Smit, Sebastiaan C. Goulooze, Roger J. M. Brüggemann, Catherine M. Sherwin, Catherijne A. J. Knibbe

**Affiliations:** 1 Department of Systems Biomedicine and Pharmacology, Leiden Academic Centre for Drug Research, Leiden University, Leiden, The Netherlands; 2 Pediatric Pharmacology and Pharmacometrics, University Children’s Hospital (UKBB), Basel, Switzerland; 3 Department of Pharmacy, Radboud Institute for Health Sciences, Radboudumc, Nijmegen, The Netherlands; 4 Department of Pediatrics, Wright State University Boonshoft School of Medicine/Dayton Children’s Hospital, Dayton, Ohio USA; 5 Department of Clinical Pharmacy, St. Antonius Hospital, Koekoekslaan 1, 3435 CM Nieuwegein, The Netherlands

**Keywords:** obesity, pediatrics, pharmacokinetics, vancomycin

## Abstract

Vancomycin is an effective but potentially nephrotoxic antibiotic commonly used for severe infections. Dosing guidelines for vancomycin in obese children and adolescents with or without renal impairment are currently lacking. This study describes the pharmacokinetics of vancomycin in a large pediatric cohort with varying degrees of obesity and renal function to design practical dosing guidelines for this population. A multi-center retrospective population pharmacokinetic study was conducted using data from patients aged 1−18 years who received >1 dose of vancomycin and had ≥1 vancomycin concentration measured between January 2006 and December 2012. Besides pharmacokinetic data, age, gender, body weight, creatinine clearance (CL_cr_, bedside Schwartz equation), ward, race, and neutropenic status were collected. Population pharmacokinetic analysis and simulations were performed using NONMEM7.4. A total of 1892 patients (5524 samples) were included, with total body weight (TBW) ranging 6−188 kg (1344 normal weight, 247 overweight, and 301 obese patients) and CL_cr_ down to 8.6 mL/min/1.73 m^2^. The two-compartment model, with clearance (CL) significantly increasing with TBW and CL_cr_, central and peripheral volume of distribution and inter-compartmental clearance increasing with TBW, performed well for all age, weight, and renal function ranges. A dosing guideline is proposed that integrates body weight and CL_cr_ resulting in effective and safe exposures across all ages, body weight, and renal functions in the pediatric population. We have characterized the full pharmacokinetic profile of vancomycin in obese children and adolescents aged 1−18 years and propose a practical dosing guideline that integrates both body weight and renal function.

**Supplementary Information:**

The online version contains supplementary material available at 10.1208/s12248-021-00577-x.

## INTRODUCTION

Over the past decades, the prevalence of childhood obesity has increased at an alarming rate. Where childhood obesity practically did not exist approximately 50 years ago, 41 million children under 5 years of age were considered overweight or obese in 2014 (1). In the USA, approximately 20% of children aged 5–18 years are considered obese (2). Pediatric obesity is typically defined using growth charts with age- and sex-specific values for the body mass index (BMI). The Centers for Disease Control and Prevention (CDC) define overweight and obesity as a BMI in the 85^th^−95^th^ percentile or above the 95^th^ percentile of these charts, respectively (3). As a result, clinicians frequently prescribe medication to children who are overweight.

It has been shown for adults that obesity can impact drug pharmacokinetics by altering different physiological processes, such as cardiac output, renal and hepatic perfusion, and function of drug-metabolizing or transporting enzymes (4,5). These principles presumably also apply to obese children, although well-designed studies that explore this are scarce (5,6). Children are generally underrepresented during drug development trials, and, if children are included, often there is no active inclusion of obese children (7). Consequently, drug labels do not provide information on drug dosing in obese children, and specific guidelines for drug dosing in pediatric obesity are currently scant (7). Clinical trials in obese children can be methodologically challenging since age- and obesity-related influences are both reflected in a child’s body weight; i.e., body weight can increase as a result of growth and development (weight for age), and of overweight or obesity (excess weight) (8). Pharmacokinetic trials in pediatric obesity should ideally include an in-depth analysis that allows for the study of the distinct influence of maturation versus overweight on drug pharmacokinetics (9), as has been demonstrated for busulfan, midazolam, and metformin (10–12).

Vancomycin is a glycopeptide antibiotic that is widely used in serious gram-positive infections including those with beta-lactam resistant *Staphylococcus aureus* and is known for its potential nephrotoxic side effects. It has been well established that vancomycin efficacy and nephrotoxicity closely relate to the 24-h area under the curve (AUC_24_) in relation to the minimal inhibitory concentration (MIC) (13). An AUC_24_/MIC threshold of 400, corresponding to an AUC_24_ of ≥400 mg*h/L assuming a MIC of 1 mg/L, has been well defined as an efficacy target, which is predominantly based on *S. aureus* infections in adults but can also be applied to children (13). In adults, an increased risk of nephrotoxicity has been observed with exposures above 677 up to 1300 mg*h/L (14,15). As such, a leading consensus guideline from infectious disease specialists, hospital pharmacists, and pediatricians from the US advocate an AUC_24_ target window of at least 400 mg*h/L up to 600–800 mg*h/L to be used in children to maximize efficacy while minimizing the risk of nephrotoxicity (13). For the current study, we translated this to a target AUC_24_ window of 400–700 mg*h/L.

Dosing of vancomycin in normal weight children has been investigated thoroughly (13). However, despite its extensive use, there is to date limited data on how to tailor the dose in obese children and adolescents (13,16). Some small retrospective studies have shown that with the same mg/kg dosing, higher trough concentrations are seen in obese children (17–19), although other studies contradict these results (20,21). None of these studies has reported on the relationship between trough concentrations and AUC_24_, which is relevant since trough concentrations are routinely measured while it is known that the relation between trough concentrations and AUC_24_ depends on age and the dosing interval (22,23). The limited number of pharmacokinetic studies conducted has proposed different covariates for vancomycin clearance in obese children and adolescents. Among others, body size descriptors like total body weight (TBW), body surface area (BSA), or fat-free mass (FFM), age, or parameters representing the renal function such as serum creatinine or creatinine clearance (CL_cr_) have been suggested (24–27).

Hence, for obese children and adolescents, current evidence suggests that the usual pediatric vancomycin dosages should be adjusted. However, the optimal dosing strategy to ensure an AUC_24_ 400–700 mg*h/L in obese children and adolescents yet remains to be established, particularly when these obese children suffer from renal dysfunction. This study characterizes the population pharmacokinetics of vancomycin in a large, multi-center clinical population of normal weight, overweight, and obese children and adolescents, with varying renal function, to design practical dose recommendations for this population.

## MATERIALS AND METHODS

### Patients and Setting

This retrospective, pharmacokinetic study was conducted using data from twenty-one hospitals of the Utah, USA–based HMO Intermountain Healthcare organization. We selected all patients aged 1–18 years who had at least two vancomycin administrations, at least one vancomycin concentration measured, and at least one weight measurement registered between start and end of treatment with vancomycin. According to local clinical practice, vancomycin dosage and concentration measurements were left to the discretion of the treating physician. Generally, vancomycin was dosed as 15 to 20 mg/kg, administered two, three, or four times per day as a 60-min infusion. Dosing adjustments were made based on therapeutic drug monitoring (TDM) blood samples which were collected as part of routine medical care. Samples could be drawn within 30 min before the dose (trough concentration), 30 min after the end of the intravenous infusion (peak concentration), or at other time points. Patients that received renal replacement therapy or extracorporeal membrane oxygenation during hospital admission were excluded from the analysis. The study was reviewed and approved by the Intermountain Healthcare and University of Utah Institutional Review Boards, and a waiver of informed consent was granted.

### Data Collection

Data on demographics, lab values, and clinical PK data were extracted from the Intermountain Healthcare system enterprise data warehouse at the University of Utah between January 1, 2006, and December 31, 2012. Data were excluded from the analysis when date and times of drug administration or drug concentrations were unavailable, where in case of missing dose amounts in less than 20% per individual, these were imputed using the last known administered amount.

Vancomycin serum drug concentrations were quantified using immunoassay via the Abbott Architect System. Assay validation was performed for clinical purposes. The linear range for the assay was 1.1 to 100 mg/L, and the limit of quantitation was 1.1 μg/mL. The intraday and interday relative standard deviations ranged from 4.7 to 7.1%.

Individuals were categorized as begin normal weight, overweight, or obese according to the WHO and CDC growth charts, where overweight and obesity were defined as >85^th^ percentile or >95^th^ percentile of the BMI (corrected for age and sex) growth charts of the WHO for age 1–2 years, and CDC for 2–18 years (3,28,29). Available covariates included age, total body weight (TBW), length, body surface area (BSA), fat-free mass (FFM), gender, race, ICU stay, serum creatinine, absolute neutrophil count, absolute lymphocyte count, and C-reactive protein (CRP). As a separate approach to investigate the influence of body weight on vancomycin pharmacokinetics, we attempted to distinguish the influence of growth-related and obesity-related changes in weight. To do this, for each patient, body weight related to growth (WT_for age and length_) and excess body weight (WT_excess_) were calculated according to equations S1 and S2 in the supplementary file, adapted from Van Rongen et al. (10). Within an individual, missing creatinine values were imputed using a next-observation-carried-backward strategy where typical values were imputed using the equation from Ceriotti et al. in case no creatinine values were available for an individual (15.6% of the individuals) (30). CL_cr_ was estimated using the bedside Schwartz equation and was studied both expressed in mL/min/1.73 m^2^ (31) and deindexed by multiplication with BSA/1.73 (CL_cr_di_), according to Eqs. (1) and (2):

1 $$ \mathrm{CLcr}\ \left(\mathrm{in}\ \mathrm{m}\mathrm{L}/\min /1.73\ \mathrm{m}2\right)=0.41\ast \mathrm{length}\ \left(\mathrm{in}\ \mathrm{cm}\right)/\mathrm{serum}\ \mathrm{creatinine}\ \left(\mathrm{in}\ \mathrm{m}\mathrm{g}/\mathrm{dL}\right) $$

2 $$ \mathrm{CLcr}\_\mathrm{di}\ \left(\mathrm{in}\ \mathrm{m}\mathrm{L}/\min \right)=\mathrm{CLcr}\ast \mathrm{body}\ \mathrm{surface}\ \mathrm{area}\ \left(\mathrm{BSA},\mathrm{in}\ \mathrm{m}2\right)/1.73 $$

More details regarding available covariates can be found in the supplementary file (methods—pharmacokinetic analysis).

### Population Pharmacokinetic Analysis

Log-transformed vancomycin serum concentrations were analyzed using non-linear mixed-effects modelling (NONMEM v7.4). Model development was performed in two steps: (1) development of the structural and statistical model and (2) a covariate analysis. In both steps, models were compared using the objection function value (OFV), where lower values indicate a better fit. In addition, goodness-of-fit plots and several other model diagnostics were considered. The final model was internally validated by normalized prediction distribution errors (NPDE) and prediction and variability corrected visual predictive check (pvcVPC). The parameter precision of the structural and final model was analyzed by the sampling importance resampling (SIR) procedure (32). The details of the pharmacokinetic analysis can be found in the supplementary file (methods—pharmacokinetic analysis).

### Dose Simulations

To evaluate existing dosing guidelines and, if necessary, design a new guideline, concentration-time profiles were simulated for several typical individuals from the dataset with different ages, body weight, and renal functions using the ranges found across the dataset. Dosing guidelines from the Infectious Diseases Society of America, the American Society of Health-System Pharmacists, the Pediatric Infectious Diseases Society and the Society of Infectious Diseases Pharmacists (abbreviated to IDSA) (13), the Dutch Pediatric Formulary (33), and the British National Formulary for Children (BNFc) (34) were evaluated (see supplementary file). Based on the final model, a dosing guideline aiming for an AUC_24_ of 400–700 mg*h/L at day 3 after the start of treatment (AUC_day3_) as a primary target was developed. The secondary target was an AUC_24_ in the first 24 h (AUC_day1_) within 400–700 mg*h/L. Lastly, trough (*C*_min_) concentrations corresponding to the primary target were explored.

## RESULTS

**Fig. 1:**
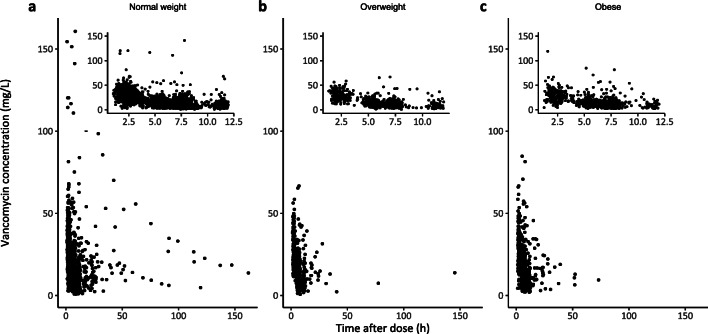
Observed vancomycin concentrations in mg/L versus time after dose in hours for **a** non-obese, **b** overweight, and **c** obese individuals. Inserts show the same data for the time frame 0 to 12 h after the last dose

Data was obtained for 1924 individuals, after which patients on renal replacement therapy or extracorporeal membrane oxygenation (*n* = 26) or without a recorded body weight (*n* = 6) were excluded. This resulted in 1892 patients in which 5524 vancomycin concentrations were available for analysis (Fig. 1). Of these patients, 247 (13%) and 301 (16%) individuals fulfilled the criteria for overweight and obesity, respectively, resulting in a broad range of body weights from 6–188 kg. Figure 1 shows the wide scatter in sampling time after dose for the three groups. Most characteristics, including age and renal function, were similarly distributed across the three weight groups (Table I). There was a broad range in CL_cr_ (bedside Schwartz equation) with values as low as 8.6 mL/min/1.73 m^2^. In total, 12 patients had a CL_cr_ under 30 mL/min/1.73 m^2^, of which 5 patients were overweight or obese. All relevant baseline characteristics are shown in Table I.

**Table I:** Baseline Characteristics

Characteristic	Normal weight (*n* = 1344)	Overweight (*n* = 247)	Obese (*n* = 301)
Age (years)	6.9 [2.9–13.2] (1.0–18.0)	7.2 [3.0–12.6] (1–17.6)	6.9 [2.5–13.2] (1.0–18.0)
Age group (*N* (% of the total of age group))	1–2 years: 214 (66)	1–2 years: 41 (13)	1–2 years: 68 (21)
	2–12 years: 727 (72)	2–12 years: 137 (14)	2–12 years: 135 (14)
	12–18 years: 403 (71)	12–18 years: 69 (12)	12–18 years: 98 (17)
Gender (% male)	57.3	53.4	54.1
Race (*N* (% of the total of the group)	Caucasian: 1198 (72)	Caucasian: 217 (13)	Caucasian: 248 (15)
	Asian: 11 (85)	Asian: 2 (15)	Asian: 0 (0)
	Hispanic: 17 (65)	Hispanic: 1 (4)	Hispanic: 8 (31)
	African American: 27 (62)	African American: 8 (19)	African American: 8 (19)
	Other: 91 (62)	Other: 19 (13)	Other: 37 (25)
TBW (kg)	20.6 [13.0–38.8] (5.8–82.6)	25.0 [13.8–51.4] (7.3–99.3)	30.0 [14.0–78.1] (7.5–188.0)
Height (cm)	119 [92–150] (62–203)	115 [87–149] (63–193)	116 [85–159] (54–196)
BMI (kg/m^2^)	16.1 [14.6–17.7] (8.6–25.6)	18.9 [17.9–23.2] (16.9–23.2)	23.2 [19.7–29.8] (18.1–60.1)
BSA (m^2^)	0.83 [0.58–1.28] (0.32–2.10)	0.89 [0.58–1.45] (0.36–2.31)	1.00 [0.57–1.86] (0.35–3.04)
Serum creatinine (mg/dL)	0.40 [0.30–0.54] (0.06–8.20)	0.40 [0.30–0.57] (0.10–3.53)	0.44 [0.30–0.61] (0.12–3.16)
Bedside Schwartz creatinine clearance (mL/min/1.73 m^2^)	121.2 [101.2–144.6] (8.6–963.5)	114.7 [91.0–142.3] (14.8–291.1)	111.7 [91.3–134.5] (21.3–323.9)
Bedside Schwartz group^*a*^ (*N* (% of the total of the group))	>90: 1100 (73)	>90: 186 (12)	>90: 220 (15)
	60–90: 200 (63)	60–90: 48 (15)	60–90: 68 (22)
	30–60: 37 (64)	30–60: 9 (16)	30–60: 12 (21)
	<30: 7 (58)	<30: 4 (33)	<30: 1 (8)
Patients admitted to ICU (%)	466 (35)	91 (37)	113 (38)
Patients with neutropenia (*N* (% of total))	223 (17)	53 (22)	40 (13.3)
No. of samples (*N* (% of total))	3968 (72)	698 (13)	858 (16)
No. of samples per individual	4 [2–7] (1–37)	4 [2–6] (1–34)	4 [2–7] (1–34)
Sampling time after dose (h)	5.8 [4.3–7.5] (1.0–162.0)	5.9 [4.5–7.6] (1.3–145.3)	6.4 [4.8–7.7] (1.3–73.0)

Values are shown as median [interquartile range] (range) unless specified otherwise

^*a*^Schwartz group is shown in mL/min/1.73 m^2^

*BMI* body mass index, *BSA* body surface area

### Population Pharmacokinetic Analysis

A two-compartment model with inter-individual variability on clearance (CL) and peripheral volume of distribution (V2) with a proportional residual error model best described the data. The pharmacokinetic parameters of the structural model without covariates are shown in Table II. For additional details regarding the population pharmacokinetic analysis, we refer to the supplementary file (results—pharmacokinetic analysis)

**Table II:** Population Pharmacokinetic Model Parameters of the Structural Base Model (Without Covariates) and the Final Model (with Covariates) for Vancomycin in Normal Weight, Overweight, and Obese Children and Adolescents Aged 1–18 Years Old with and without Renal Impairment

Parameter	Structural model (RSE %)	Final model (RSE %) [95% CI]
Fixed effects		
CL (L/h)	2.17 (2)	-
TVCL $\times {\left(\frac{\mathbf{TBW}}{\mathbf{22.1}}\right)}^{\boldsymbol{\uptheta} \mathbf{1}}\times \left(\frac{{\mathbf{SCHW}}^{\boldsymbol{a}}}{\mathbf{100}}\right)$		
TVCL (L/h)	-	2.12 (1) [2.07–2.17]
θ_1_	-	0.745 (2) [0.720–0.768]
V1 (L)	5.27 (8)	-
TVV1 $\times \left(\frac{\mathbf{TBW}}{\mathbf{22.1}}\right)$		
TVV1 (L)	-	8.90 (3) [8.50–9.33]
Q (L/h)	2.24 (4)	-
TVQ $\times {\left(\frac{\mathbf{TBW}}{\mathbf{22.1}}\right)}^{\boldsymbol{\uptheta} \mathbf{2}}$		
TVQ (L)	-	1.55 (5) [1.44–1.65]
θ_2_	-	0.599 (9) 0.517–0.685]
V2 (L)	11.9 (8)	-
TVV2 $\times \left(\frac{\mathbf{TBW}}{\mathbf{22.1}}\right)$		
TVV2 (L)	-	12.3 (6) [11.2–13.6]
Inter-individual variability (IIV, %) b^*,*^c		
CL	52.8 (3)	28.7 (5) [27.1–30.7]
Covariance IIV _CL-V2_	-	−0.085 [−0.11 to −0.062]
V2	89.4 (7)	110 (7) [95.9–130]
Residual variability		
Proportional errord^*,*^e	0.107 (7)	0.0789 (6) [0.0746–0.0836]
OFV	−1886.4	−5222.5

^*a*^Schwartz value is maximized to 120 mL/min/1.73 m^2^

^*b*^Shrinkage of inter-individual variability in the final model is 24% for CL, 57 % for V2

^*c*^Coefficient of variation, calculated by $\sqrt{\left({\boldsymbol{e}}^{{\boldsymbol{\omega}}^{\mathbf{2}}}-\mathbf{1}\right)}$

^*d*^Proportional error is shown as σ

^*e*^Epsilon shrinkage for the final model is 16%

*CI* confidence interval obtained from sampling importance resampling (SIR) procedure, *CL* clearance, *OFV* objective function value, *Q* inter-compartmental clearance between V1 and V2, *RSE* relative standard error based on the covariance step in NONMEM, *SCHW* creatinine clearance based on bedside Schwartz equation, *TBW* total body weight, *TVCL* typical value of CL for an individual weighing 22.1 kg and with creatinine clearance of 100 mL/min/1.73 m^2^, *TVQ* typical value of Q for an individual weighing 22.1 kg, *TVV1* typical value of V1 for an individual weighing 22.1 kg, *TVV2* typical value of V2 for an individual weighing 22.1 kg, *V1* volume of distribution of central compartment, *V2* volume of distribution of the peripheral compartment

**Fig. 2:**
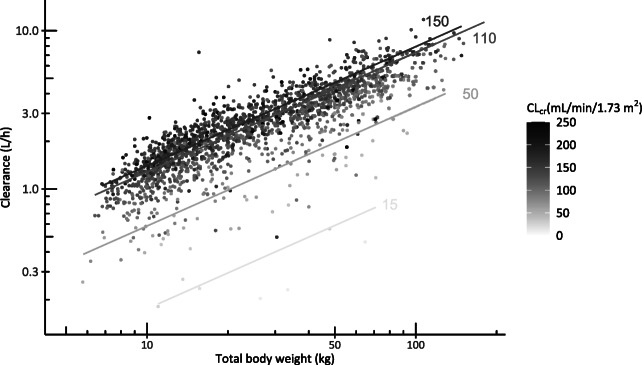
Vancomycin clearance (in L/h) versus total body weight (in kg) for varying creatinine clearance values (CL_cr_). Each dot represents one individual, with darker color representing a higher CL_cr_. The lines show how clearance changes with body weight over the available weight range according to the final model for four typical values of CL_cr_ (i.e., 15, 50, 110, and 150 mL/min/1.73 m^2^), with corresponding CL_cr_ value shown in the figure for each line (mL/min/1.73 m^2^). *CL*_*cr*_ creatinine clearance based on the bedside Schwartz equation (in mL/min/1.73 m^2^)

**Fig. 3:**
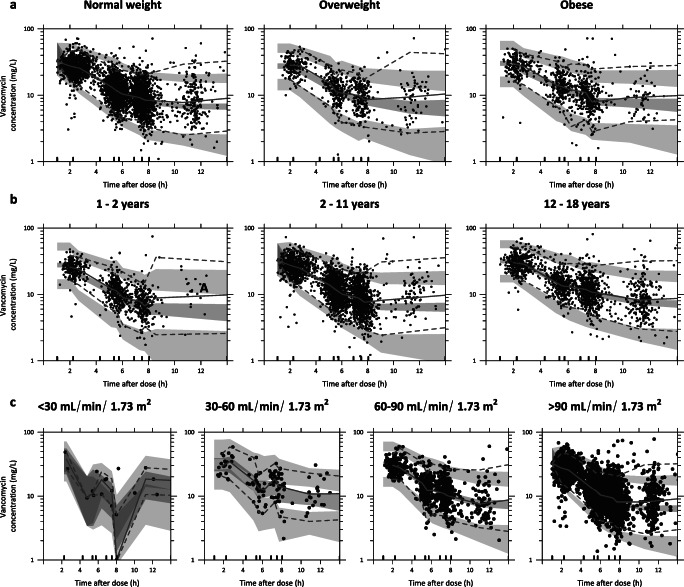
Prediction and variability corrected visual predictive check (pvcVPC), split for **a** weight group, **b** age group, and **c** for renal function group based on bedside Schwartz. Prediction-corrected observations are shown as dots, with the median, 2.5th and 97.5th percentiles shown as solid, lower, and upper dashed lines. Grey shaded areas represent the 95% confidence intervals of the median (dark grey) and 2.5th and 97.5th percentiles (light grey) of predicted concentrations (*n* = 500) based on the pharmacokinetic model. Intervals of the bins are shown by the vertical ticks on the bottom of the plot

In the covariate analysis, we found that vancomycin CL was best described using both CL_cr_, capped at 120 mL/min/1.73 m^2^ (linear function) and TBW (power equation with estimated exponent 0.745 (95% CI 0.720–0.768)). The specific influence of TBW and CL_cr_ on vancomycin CL is visualized in Fig. 2. In addition, TBW was the most significant covariate for central and peripheral volume of distribution and inter-compartment clearance. The pvcVPC plots (Fig. 3) based on the final pharmacokinetic model, split for several subpopulations, i.e., underweight, normal weight, and overweight, different age groups, and varying renal function, indicate a good predictive performance across all subgroups with a good agreement between simulated and observed data. The goodness-of-fit and validity of the model were confirmed by goodness-of-fit plots (Figure S1, supplementary file) and NPDE (Figure S2, supplementary file). Only for the group with the lowest renal function (<30 mL/min/1.73 m^2^), some over-prediction is seen on the goodness-of-fit plots, which may result from the small number of individuals (*n* = 12) across different subpopulations. The final pharmacokinetic model parameters are shown in Table II, for which a NONMEM control stream can be found in the supplementary file.

### Dose Simulations and Proposed Dosing Guideline

**Fig. 4:**
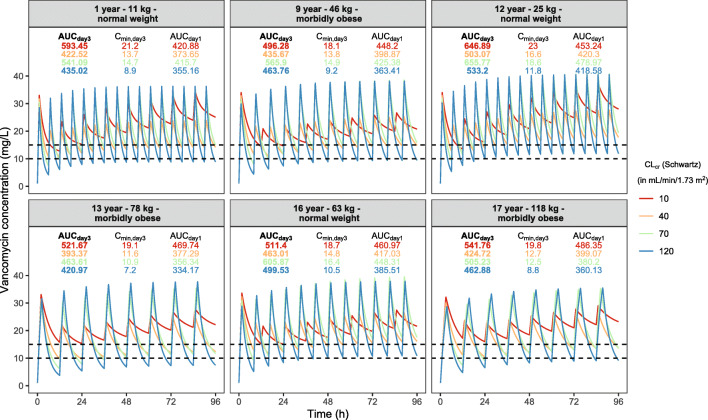
Vancomycin concentrations (mg/L) versus time (hours) in different typical individuals with body weight ranging 10–120 kg and renal function ranging 10–120 mL/min/1.73 m^2^ where vancomycin is dosed according to the proposed dosing guideline (Table III). For each individual, AUC (in bold), *C*_min_ at day 3, and AUC at day 1 are shown in the graph (where color corresponds to the individual's renal function). Dashed lines represent the target concentrations for the trough concentrations (10–15 mg/L). *AUC* area under the curve, *C*_*min*_ minimum (trough) concentration

Based on the final model in which the influence of both renal function and body weight on vancomycin clearance and weight on volume of distribution were quantified, we defined dosing recommendations for the pediatric population (Table III). As shown in Table III, the first dose is 15 mg/kg for all groups followed by doses adjusted to body weight and renal function. The obtained concentration-time profiles using this dosing guideline for six representative individuals from the dataset (normal weight and morbidly obese individuals ranging from 1 to 17 years and 11 to 118 kg) are shown in Fig. 4. For each individual, four curves with different renal functions (bedside Schwartz 10–120 mL/min/1.73 m^2^) are shown. For reference, the profiles for the same individuals using the currently leading pediatric dosing guidelines are shown in Figure S3 in the supplementary file. When the proposed dose nomogram is used, the obtained AUC_day3_ (defined as the AUC from 48 to 72 h after the first dose) was within the target of 400–700 mg*h/L for all individuals, regardless of body weight, weight group (obese or normal weight), renal function, or age. Additionally, already in the first 24 h target, AUCs were reached in all individuals, except for the individuals with renal function >120 mL/min/1.73 m^2^ (Fig. 4). Similar results are obtained when the dosing guideline is adapted to a continuous infusion dosing regimen (Figure S4 in the supplementary file). Here, 15 mg/kg is given as a loading dose, followed after 3 h by the proposed daily dose given as a 24-h infusion. For the reader’s convenience, we have provided this continuous infusion dosing guideline in the supplementary file (Table S1). The results obtained using the dosing guideline as shown in Table III and Fig. 4 contrast with what was obtained using the currently leading dosing guidelines (IDSA, Dutch Pediatric Formulary, BNFc), as shown in Figure S3, where the current guidelines result in high, potentially toxic exposures (AUC_day3_ >700 mg*h/L) especially in children with renal impairment or who are considered obese. This particularly applies to BNFc and IDSA guidelines, which do not recommend dose adjustments for patients with reduced renal function. Figure 4 shows that for the typical individuals, trough concentrations corresponding to an AUC_day3_ 400–700 mg*h/L vary between 7.2 and 23 mg/L, when dosed according to the proposed dosing guideline in Table III.

**Table III:** Dosing Guideline for Intermittent Dosing of Vancomycin in Children and Adolescents Aged 1–18 years Based on Total Body Weight and Renal Function According to Bedside Schwartz

Bedside Schwartz creatinine clearance (mL/min/1.73 m^2^)	Total body weight (kg)			Relative daily dose (%)
	<30	30–70	>70	
>90	15 mg/kg every 6 h	15 mg/kg every 8 h	18 mg/kg every 12 h	100%
50–90	11 mg/kg every 6 ha	11 mg/kg every 8 ha	12 mg/kg every 12 ha	70%
30–50	5 mg/kg every 6 ha	5 mg/kg every 8 ha	6 mg/kg every 12 ha	35%
10–30	5 mg/kg every 12 ha	3 mg/kg every 12 ha	3 mg/kg every 12 ha	15%

^*a*^First dose is 15 mg/kg

## DISCUSSION

In this study, we provide a practical dosing guideline for children and adolescents with varying levels of obesity and renal function based on a thorough characterization of the vancomycin pharmacokinetics in a large pediatric and adolescent population aged 1–18 years that consists of normal weight, overweight, and obese individuals with a wide range of renal functions. We have demonstrated that vancomycin clearance can be well predicted using a combination of renal function calculated by the bedside Schwartz formula and total body weight. To our best knowledge, the pediatric pharmacokinetics of vancomycin has not been described before in such a large and rich dataset, with a broad range and overlay of multiple relevant covariates such as age, body weight, and renal function and where the vancomycin samples showed a good distribution in time after dose, especially over the first 12 h. This straightforward dosing guideline is in line with the IDSA vancomycin dose recommendation for non-obese children (15 mg/kg four times daily) (13) and our recently proposed dose recommendations for vancomycin in obese adults (35 mg/kg per day) (35). This means that dose recommendations in this guideline are the same as the leading IDSA guideline for children and adolescents with a normal renal function in case of a body weight <30 kg (13), while being similar to the recently proposed obese adult dose recommendations in case of a body weight >70 kg (35). However, it adds dose adaptations for pediatric obesity and, in both normal weight and overweight/obese children, for renal impairment. The dosing guideline for obese adults includes a dose cap when the total daily dose reaches 5500 mg (35). Although such a maximum daily dose is expected to be reached in cases of extreme obesity only, we recommend to use this dose cap in the pediatric population as well.

We demonstrate that by following our proposed dosing guideline (Table III), effective exposures with minimal risk of toxicity (AUC_day3_ between 400 and 700 mg*h/L) can be expected throughout the population. Besides, by starting with a first (loading) dose of 15 mg/kg in all groups, target exposures can be reached in the first 24 h after the start of treatment for most individuals, both in intermittent or continuous infusion regimens. The simulations presented in this study serve the purpose to illustrate what can be expected in terms of vancomycin levels for different individuals (including extremes) in the target population when the new dosing guideline is used. These simulations do not include remaining random variability, but as this variability is well-known for vancomycin, being the primary reason why vancomycin TDM is advised as part of standard practice (13), we considered this random variability out of scope for the current manuscript. In light of these TDM recommendations, we show that trough concentrations may vary vastly, with values ranging from 7.2 to 23 mg/L in our typical individuals, even though the exposure is within the target for these individuals (Figure 4). The variability in trough concentrations related to target exposure as a result of dosing frequency, age, or weight has been described before for several populations, including obese adults and normal weight children (22,23,35). Therefore, clinicians should not base dose adjustments on trough concentrations alone, but preferably use Bayesian forecasting to relate TDM samples to predict exposure, as is also recommended in the recently revised vancomycin therapeutic drug monitoring guideline (13). For Bayesian forecasting, the current PK model can be used as a basis.

There is currently a limited number of vancomycin pharmacokinetic studies that have been performed in obese children or adolescents (18,19,24–27). In contrast to our study, the majority of these publications lack specific dose recommendations, in particular regarding the combination of renal impairment and obesity. Several studies found that when vancomycin was dosed on a similar mg/kg basis in obese and non-obese children, higher trough concentrations were obtained in obese children (16,19,24,18). This finding is in agreement with our observations, showing that the IDSA and BNFc guidelines lead to increasing exposure and trough concentrations with increasing body weight to the point where the dose is being capped. Most pharmacokinetic studies found that clearance increases with body weight, but varying covariate relationships have been described. An analysis by Lanke et al. in 463 adolescents aged 12–18 years found that vancomycin clearance increased with TBW and creatinine clearance based on the bedside Schwartz equation, similar to our results (25). Another study in 196 mostly adolescent overweight and obese children found that besides serum creatinine, fat-free mass best predicted vancomycin clearance (26). In their dataset, total body weight could not be identified as a predictor of clearance. It is unclear what explains these results, but it cannot be excluded that these findings are explained by the absence of adolescents with normal weight unlike the data of our study. Lastly, Le et al. have also found that in 87 pairs of obese and non-obese children, aged 2–18 years, vancomycin clearance can best be predicted by a combination of total body weight (using an allometric function with exponent 0.75), serum creatinine, and age (27), which is roughly in line with our results. However, the authors state that the differences between obese and non-obese individuals are small and do not necessitate any dose adjustments. Our study clearly shows that dose adjustments are however necessary to prevent subtherapeutic or toxic exposures.

In the covariate analysis, we have investigated several approaches for the inclusion of weight and renal function as covariates for vancomycin clearance. Regarding renal function, we found that the use of the bedside Schwartz equation outperformed the use of serum creatinine or creatinine ratio for predicting vancomycin clearance. It should be recognized that the performance of this equation in the obese pediatric population is uncertain (36). In recognition of this limitation, we have tried several empirical approaches to identify the best renal function estimate to predict vancomycin clearance, among which are the serum creatinine, creatinine ratio, de-indexation of the Schwartz equation, or re-estimation of *k* in the bedside Schwartz equation. In all these cases, the bedside Schwartz equation outperformed the other approaches in predicting vancomycin clearance. This might lie in the fact that bedside Schwartz is normalized for body surface area (as it is expressed as mL/min/1.73 m^2^) and is, as a consequence of this normalization, not dependent on body weight. In addition, we found that capping CL_cr_ to 120 mL/min/1.73 m^2^ leads to a better model fit than without capping of CL_cr_ or when capping at higher values. Hence, our data indicates that even though overweight or obese children might show some indication of hyperfiltration based on the Schwartz equation, this does not translate to an increased vancomycin clearance.

As for the use of weight in predicting clearance, it is important to realize that in pediatrics, weight can be a result of either growth (reflecting age) or excessive growth (in obesity). In this perspective, we did not only investigate the influence of body weight as a variable but also looked into more sophisticated models that separately characterize the influence of weight for age-and-length and weight excess following (equation S8 in the supplementary file). In our study, there was no benefit of these models over a simple covariate model using only total body weight. This implies that for vancomycin clearance in children, there seems to be no difference in the influence of weight resulting from growth and development and excess weight resulting from obesity. Our results are in line with studies with similar populations for metformin and midazolam, where for metformin clearance, midazolam volume of distribution, and busulfan clearance, WT_for age and length_/WT_excess_ models or a model using the Z-score as covariate performed similarly as compared to a model with TBW as a covariate (10–12). In addition, we could not identify a maturation model (using a body weight–dependent exponent following equation S7 in the supplementary file) for clearance. This is not unexpected, since it is well-known that the maturation of renal excretion processes such as glomerular filtration rate (GFR) is nearly complete around 1 year of postnatal age (37). As such, in our population of children over one-year-old, such a maturation function was not of added value. This is substantiated by a pediatric pharmacokinetic study with vancomycin, where a body weight-dependent exponent was found to be superior compared to a simple model with TBW as a covariate, since this study was done in non-obese children mostly under 1 year of age (38). As a last remark, we estimated an exponent of 0.745 for the effect of TBW on vancomycin clearance. This value is close to 0.75 which is often used for weight-based allometric scaling of pediatric drug clearance from adult values. Although the principles of allometric scaling have been well established in predicting drug clearance in normal weight children over 5 years of age, this is not the case for obese children or children aged below 5 years (39). For this reason, we decided to keep the estimated value of 0.745 in the final model, keeping in mind that we cannot rule out coincidence as the cause for finding a similar value as the allometric exponent of 0.75 in this particular population.

Some limitations of our study should be addressed. Children under 1 year of age were excluded from this study. Therefore, readers should not use our results in children below 1 year of age for which we refer to other dosing guidelines (22). In addition, although we included patients with renal function ranging down to 8.6 mL/min/1.73 m^2^, there were relatively few patients with an estimated renal function <30 mL/min/1.73 m^2^ (*n* = 12). The diagnostics of our final model show some slight underprediction of vancomycin concentrations in this group (Figure S1, supplementary file), while the dose recommendations show that due to an increased elimination half-life, steady-state concentration has not been reached on day 3 in this patient group. Consequently, our dose recommendations must be used with extra caution for this subgroup. Lastly, vancomycin was given exclusively as intermittent infusions in the population included in our dataset. With this study design, we can adequately estimate clearance, which mainly drives the maintenance dose for both intermittent and continuous regimens. However, some caution should be applied when extrapolating our results to continuous infusion regimens.

## CONCLUSIONS

We have successfully characterized the population pharmacokinetics of vancomycin in children and adolescents aged 1 year and above, with varying degrees of obesity and renal functions. Vancomycin clearance can be well predicted using a combination of CL_cr_ (using the bedside Schwartz equation) and total body weight. Using this model, we have designed a dosing guideline that provides quantitative detail on the IDSA recommendation of 15 mg/kg four times daily by specifying the dose reductions required for renal impairment in both obese and non-obese individuals. With this dosing guideline, effective and safe exposures at day 3 (AUC_day3_ of 400–700 mg*h/L), but also in the first 24 h of treatment, are expected throughout the pediatric population aged 1–18 years.

## Supplementary Information

ESM 1 (PDF 735 kb)
