# Supplementary material for: Dosing Recommendations for Vancomycin in Children and Adolescents with Varying Levels of Obesity and Renal Dysfunction: a Population Pharmacokinetic Study in 1892 Children Aged 1–18 Years
Source: AAPS J. 2021 Apr 11;23(3):53. doi: 10.1208/s12248-021-00577-x (PMC8038958; doi:10.1208/s12248-021-00577-x)
Supplement: Supplementary file 1 — (PDF 735 kb) [file 12248_2021_577_MOESM1_ESM.pdf]

Supplementary file for

Dosing recommendations for vancomycin in children and adolescents with varying levels of obesity and renal dysfunction: a population pharmacokinetic study in 1892 children aged 1 – 18 years

Cornelis Smit, Sebastiaan C. Goulooze, Roger. J.M. Brüggemann, Catherine M. Sherwin, Catherijne A. J. Knibbe

Contents:

1. Methods – pharmacokinetic analysis 2
2. Results – pharmacokinetic analysis 4
3. Pediatric dosing guidelines used for simulations 6
4. Supplementary table

Table S1. Dosing guideline for continuous infusion 7

5. Supplementary figures

Figure S1. Distribution of the normalized prediction distribution errors (NPDE) for the final model 8

Figure S2. Prediction and variability corrected visual predictive check (pvcVPC), split for the age 9

Figure S3. Prediction and variability corrected visual predictive check (pvcVPC), split for renal function 9

Figure S4. Simulations in several typical individuals according to three existing dosing guidelines 10

Figure S5. Simulations in several typical individuals for the continuous infusion dosing guideline 11

6. NONMEM control stream for the final model 12
7. References used in the supplementary file

1. Methods – pharmacokinetic analysis

Covariates

Available covariates were age, total body weight (TBW), length, gender, race, ICU-stay, serum creatinine, absolute neutrophil count, absolute lymphocyte count and C-reactive protein (CRP).

Body weight related to growth ($WT_{for\ age\ and\ length}$) and excess body weight (WT_{excess}), were calculated according to equation 1 and 2 (adapted from Van Rongen et al. (1)):

$$WT_{age\ and\ length} = BMI_{for\ age\ and\ gender} \times length^2 \quad (S1)$$

$$WT_{excess} = TBW - WT_{age\ and\ length} \quad (S2)$$

Where TBW is total body weight in kg, length in cm and $BMI_{for\ age\ and\ gender}$ is the p50 BMI value based on the gender specific WHO or CDC BMI-for-age growth charts for 1 – 2 years and 2 – 18 years, respectively (2, 3).

If a patient's height was unknown, height was imputed using the median value of the CDC height-for-age chart (2). This was done for 18 individuals, corresponding to 0.95% of the total amount of analyzed individuals.

Body Surface Area (BSA) was calculated using the Mosteller equation (4).

FFM was estimated using the equations of Al-Sallami and Peters (5, 6).

Serum creatinine, quantified using IDMS Traceable Vitros CREA Slides and the Vitros 5.1 FS Chemistry System analyzer (Ortho Clinical Diagnostics, Inc, Rochester, New York), was included when measured within 168 h before or after a vancomycin dose. All serum creatinine values within this time-window were retained in the dataset and analyzed as time-varying covariates. Within an individual, missing creatinine values were imputed using a next-observation-carried-backward strategy where typical values were imputed using the equation from Ceriotti et al. in case no creatinine values were available for an individual (15.6% of the individuals) (7). CL_{Cr} was estimated using the bedside Schwartz equation and was studied both expressed in mL/min/1.73 m^2 (8) and deindexed by multiplication with BSA/1.73 (CL_{cr_di}) , according to equations (S3) and (S4):

$$CL_{cr}\ (\text{in ml/min/1.73 } m^2) = k * \text{length (in cm)} / \text{serum creatinine (in mg/dL)} \quad (S3)$$

$$CL_{cr\_di}\ (\text{in ml/min}) = CLcr * \text{body surface area (BSA, in } m^2) / 1.73 \quad (S4)$$

Where k is a variable fixed to 0.41 in the revised bedside Schwartz equation used in this study. In addition, we also tried to re-estimate k during covariate analysis.

We also calculated the ratio between the observed and typical creatinine value for age (creatinine-ratio).

Neutropenia was defined as an absolute neutrophil count $<1.5 * 10^9$ cells/L blood.

Modeling strategy

Log-transformed vancomycin serum concentrations were analyzed using non-linear mixed-effects modelling (NONMEM v7.4, Icon Development Solutions, Ellicott City, MD, USA (9)) with Perl-speaks-NONMEM (v4.9.0) and the Pirana (v2.9.9) interface (10, 11). R (v3.6.1) and Rstudio (v1.2.1335) were used for data manipulation and visualization. Vancomycin measurements reported as being below the limit of quantification (0.7% of the observations) or drawn within 1 hour after the start of the infusion (n = 218 samples, 3.7% of the observations) were excluded. Patients were analyzed as separate individuals when age increased with ≥10% or when there was ≥14 days between vancomycin administrations. Population pharmacokinetic modelling was conducted using first-order conditional estimation with inter-individual variability assumed to be log-normally distributed. One- two- and three-compartment models with additive, proportional, or a combined error model were evaluated. Nested models were compared using the objective function value (OFV, i.e. -2log likelihood (-2LL)). For structural and statistical models, a drop ≥3.84, corresponding to a p-value of <0.05 for one degree of freedom, was considered statistically significant. Models were evaluated by inspection of goodness-of-fit plots (observed versus individual or population predicted vancomycin concentrations, conditional weighted residuals versus time after dose or population predicted vancomycin concentrations), which were split for age, weight and renal function. Lastly, the precision of parameter estimates, shrinkage, and the conditional number (ratio between the highest and lowest eigenvalue) were taken into consideration.

For the covariate analysis, potential covariates were identified based on inter-individual variability versus covariate plots. Continuous covariates were entered into the model using equation (S5) for exponential relations and (S6) for linear relations:

$$P_i = P_p \times \left(\frac{COV}{COV_{standard}}\right)^X \qquad (S5)$$

$$P_i = P_p \times (1 + Y \times (COV\text{-}\ COV_{median})\ ) \qquad (S6)$$

where P_i and P_p are the individual and population parameter estimates, COV is the covariate value, COV_{median} is the median value for the covariate. X represents the exponent for a power function, and Y is the slope parameter for the linear covariate relationship. Linear covariate relations could also be entered into the model by using equation 3 with X fixed to 1. As it has been shown that with body weight as a covariate the scaling factor X may decrease with age for clearance in children (12), for X also a body weight-dependent exponent (BDE) according to equation (S7) was tested (13, 14):

$$X = F \times TBW_i^Z \qquad (S7)$$

where TBW_i is the individual's total body weight, F is the intercept of the scaling exponent, and Z is the exponent that allows the scaling exponent to change with body weight.

A WT_{excess} covariate model was tested using equation (S8), as described earlier (1, 15):

$$P_i = P_p \times \left(\frac{WT_{age\ and\ length}}{TBW_{median}}\right)^U + (V \times WT_{excess}) \qquad (S8)$$

where P_i and P_p are the individual and population parameter estimates, $WT_{age\ and\ length}$ is the body weight related to growth (equation 1), WT_{excess} the excess body weight (equation 2), TBW_{median} is the median total body weight, U is the scaling exponent for $WT_{age\ and\ length}$ (either fixed to 0.75 or estimated), V represents the linear influence of WT_{excess} on the parameter value. Categorical covariates were entered into the model by calculating a separate pharmacokinetic parameter for each category of the covariate.

Inclusion of a covariate was justified upon assessing the OFV drop (≥10.8 points, corresponding with p <0.001) between models with or without this covariate. Also, goodness-of-fit plots were reviewed as described earlier with specific emphasis on the plots split for age (1 – 2, 2 – 12 and 12 – 18 years), estimated Cl_{cr} (<30, 30 – 60, 60 – 90 and >90 mL/min/1.73 m^2) and weight group (normal weight, overweight and obese). Lastly, it was assessed whether the inter-individual variability decreased, and if trends in the inter-individual variability versus covariate plot disappeared.

The resulting final model was internally validated by assessment of normalized prediction distribution errors (NPDE) (n = 10.000 datasets) and prediction and variability corrected visual predictive check (pvcVPC) (n = 500 datasets). These diagnostics were split for age group (1 – 2, 2 – 12 and 12 – 18 years), estimated renal function (<30, 30 – 60, 60 – 90 and >90 mL/min/1.73 m^2) and weight group (normal weight, overweight and obese) (16). Parameter precision of the structural and final model was analyzed by the sampling importance resampling (SIR) procedure (17).

2. Results – pharmacokinetic analysis

A two-compartment model with inter-individual variability on clearance (CL) and peripheral volume of distribution (V2) with a proportional residual error model best described the data.

In the covariate analysis, we found an important influence of both renal function expressed using Schwartz formula (CL_{cr}) and total body weight (TBW) on CL. Vancomycin CL was best described by linear implementation of CL_{cr} which was maximized at 120 mL/min/1.73 m^2 and a power equation for TBW (ΔOFV -2356.1 compared to the structural model without covariates [p <0.001]). Higher cut-off values or no capping of CL_{cr} lead to a significantly worse fit (ΔOFV +413.1, +15.6, +91.6, for models without capping or capping at 140 or 160 ml/min/1.73 m^2, respectively [all p <0.001], compared to a model where CL_{cr} was capped at 120 ml/min/1.73 m^2). This combined covariate model outperformed models with the separate implementation of TBW or CL_{CR}, i.e. TBW on CL with a power function (ΔOFV -1125.7 [p <0.001] compared with the structural model without covariates), TBW on CL with a body weight-dependent exponent (ΔOFV -1180.9 [p <0.001] compared with the structural model without covariates) and CL_{cr_di} on CL using a power function (ΔOFV -2230.3 [p <0.001] compared with the structural model without covariates). The model with CL_{cr} and TBW on CL was also superior over a model where TBW was combined with creatinine or creatinine-ratio as a covariate on CL, all with a power equation (ΔOFV -2126.9 or -2083.5, respectively, [both p <0.001] compared with the structural model without covariates). Re-estimating the value k in the Schwartz-equation did not significantly improve the model compared to using the standard value of 0.413 in the original bedside Schwartz-equation (ΔOFV of -4.5 [p >0.01] compared to using a fixed value of 0.413).

A covariate model that uses $WT_{age\ and\ length}$ and WT_{excess} (equation 6) instead of TBW resulted in a similar OFV and goodness-of-fit as the model with only TBW as covariate (ΔOFV -1129.2 versus ΔOFV -1125.7 compared with the structural model without covariates, respectively [p >0.01]). For the implementation of CL_{cr}, a model with an estimated exponent compared to a linear model led to similar results regarding the goodness-of-fit and OFV (estimated exponent 0.94, ΔOFV -6.1 compared to the model with a linear function with one additional degrees of freedom [p >0.01]). These results indicate that regarding the influence of body weight on vancomycin clearance, the influence of weight from growth is similar to the influence of excess weight. Inclusion of neutropenia as a binary covariate on CL did not improve the model (ΔOFV +0.8 compared to the model with TBW and CL_{cr} on CL [p >0.05]). No other covariates for CL could be identified.

Both V1 and V2 were significantly influenced by TBW in a linear function (ΔOFV -830.4 [p <0.001] compared to the model without covariates on V1 or V2). There was no significant difference between a linear or a power function with an estimated exponent for TBW (estimated exponent 1.05, ΔOFV -2.3 points compared to the model with TBW on V1 and V2 linearly, p >0.05). The model with TBW linearly on V1 and V2 provided a slightly better fit compared to a WT_{excess} model for V1 and V2 using equation (6), which resulted in an OFV reduction of -813.5 points (p >0.01). The addition of TBW exponentially on Q gave a further improvement in OFV (ΔOFV -113.4 [p <0.001]). Lastly, the covariance between CL and V2 was included in the model using an OMEGABLOCK, decreasing OFV with 36 points.

After implementing covariates, inter-individual variability on CL reduced from 52.8% in the structural model without covariates to 28.7% in the final model, and inter-individual variability on V2 slightly increased from 89.4% to 109.5% (shrinkage 57%). As the goodness-of-fit and OFV substantially deteriorated when inter-individual variability for V2 was removed from the model (ΔOFV +457.2 [p <0.001]), we decided to retain it in the final PK model.

3. Pediatric dosing guidelines used for simulations

Infectious Diseases Society of America, the American Society of Health-System Pharmacists, the Pediatric Infectious Diseases Society and the Society of Infectious Diseases Pharmacists (IDSA) (18)*:*

15 mg/kg every 6 hours (max 3600 mg / day). Obese: loading dose 20 mg/kg

Dutch Paediatric Formulary (19)*:*

15 mg/kg every 6 hours + GFR 50 – 80: every 24h, GFR 10 – 50 every 48 hours. Maximum 4 gram/day.

British National Formulary for Children (BNFc) (20)*:*

< 12y: 10 – 15 mg/kg every 6h (no maximum dose).

12 years and older: 15 mg/kg every 8 hours (maximum 2 g).

4. Supplementary Table

Table S1. Dosing guideline for continuous infusion of vancomycin in children and adolescents aged 1 – 18-years based on total body weight and renal function according to bedside Schwartz.

**Schwartz creatinine clearance (mL/min/1.73 m^2)**	**Total body weight (kg)**			**Relative daily dose (%)**
	**<30**	**30 -70**	**>70**	
**>90**	60 mg/kg over 24 h[a]	45 mg/kg over 24 h[a]	36 mg/kg over 24 h[a]	100%
**50 – 90**	44 mg/kg over 24 h[a]	33 mg/kg over 24 h[a]	24 mg/kg over 24 h[a]	70%
**30 – 50**	20 mg/kg over 24 h[a]	15 mg/kg over 24 h[a]	12 mg/kg over 24 h[a]	35%
**10 – 30**	10 mg/kg over 24 h[a]	6 mg/kg over 24 h[a]	6 mg/kg over 24 h[a]	15%

[a] Loading dose is 15 mg/kg, followed after 3 hours with proposed maintenance dose.

5. Supplementary Figures

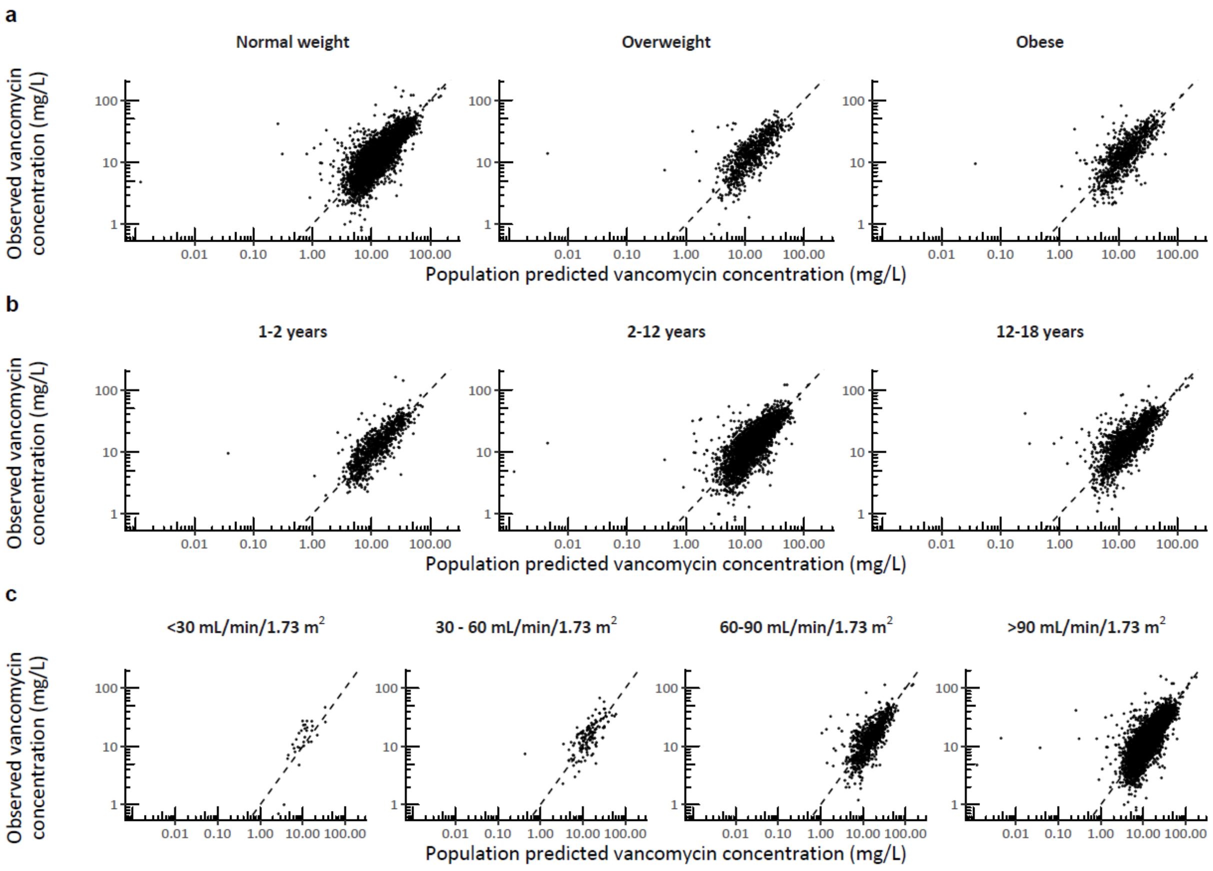

Figure S1. Observed versus population predicted vancomycin concentrations for the final model, split for (a) weight group, (b) age group or (c) renal function group (creatinine clearance based on the bedside Schwartz equation).

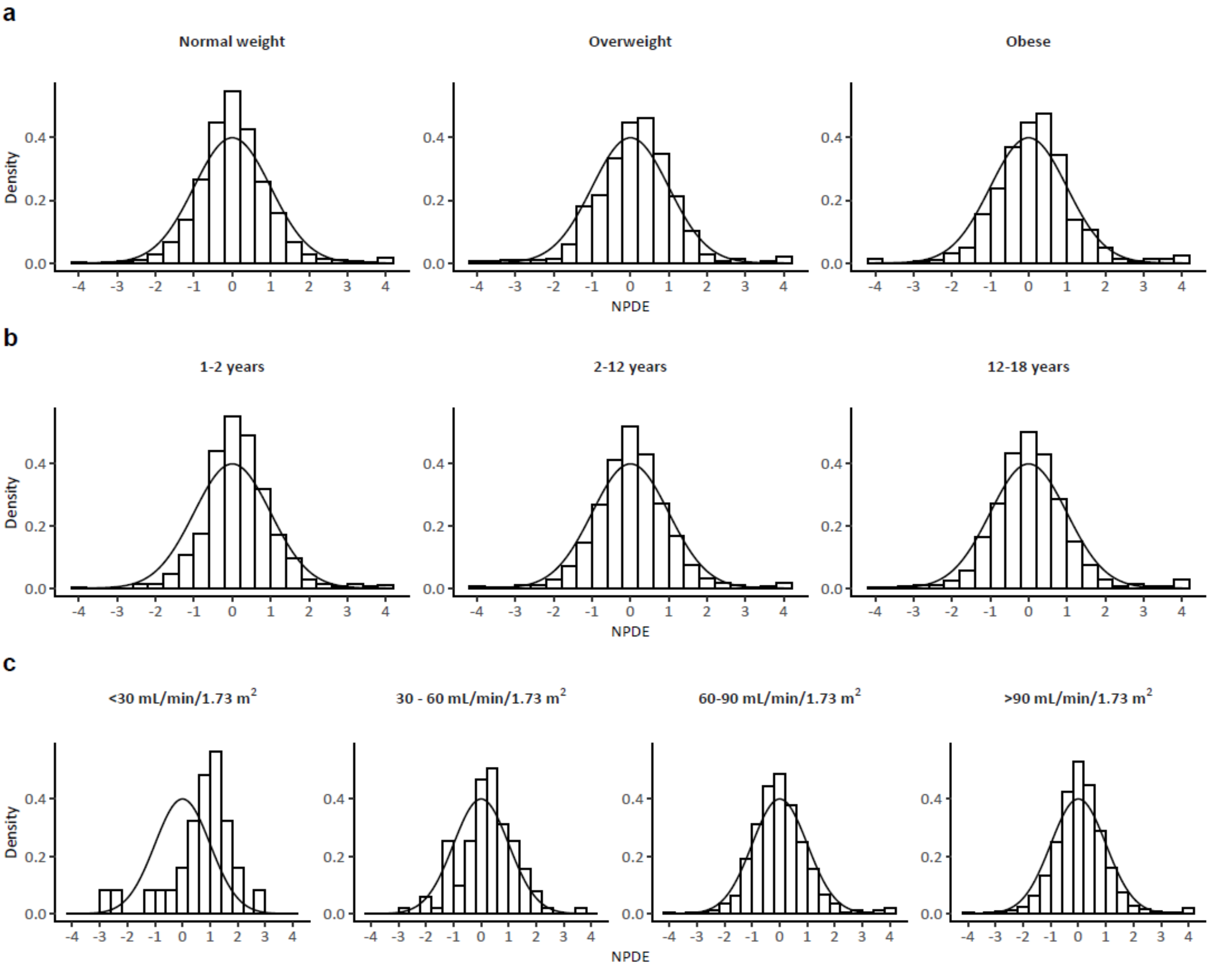

Figure S2. Distribution of the normalized prediction distribution errors (NPDE) for the final model, split for (a) weight group, (b) age group or (c) renal function group (based on the bedside Schwartz equation). The solid line depicts a normal distribution.

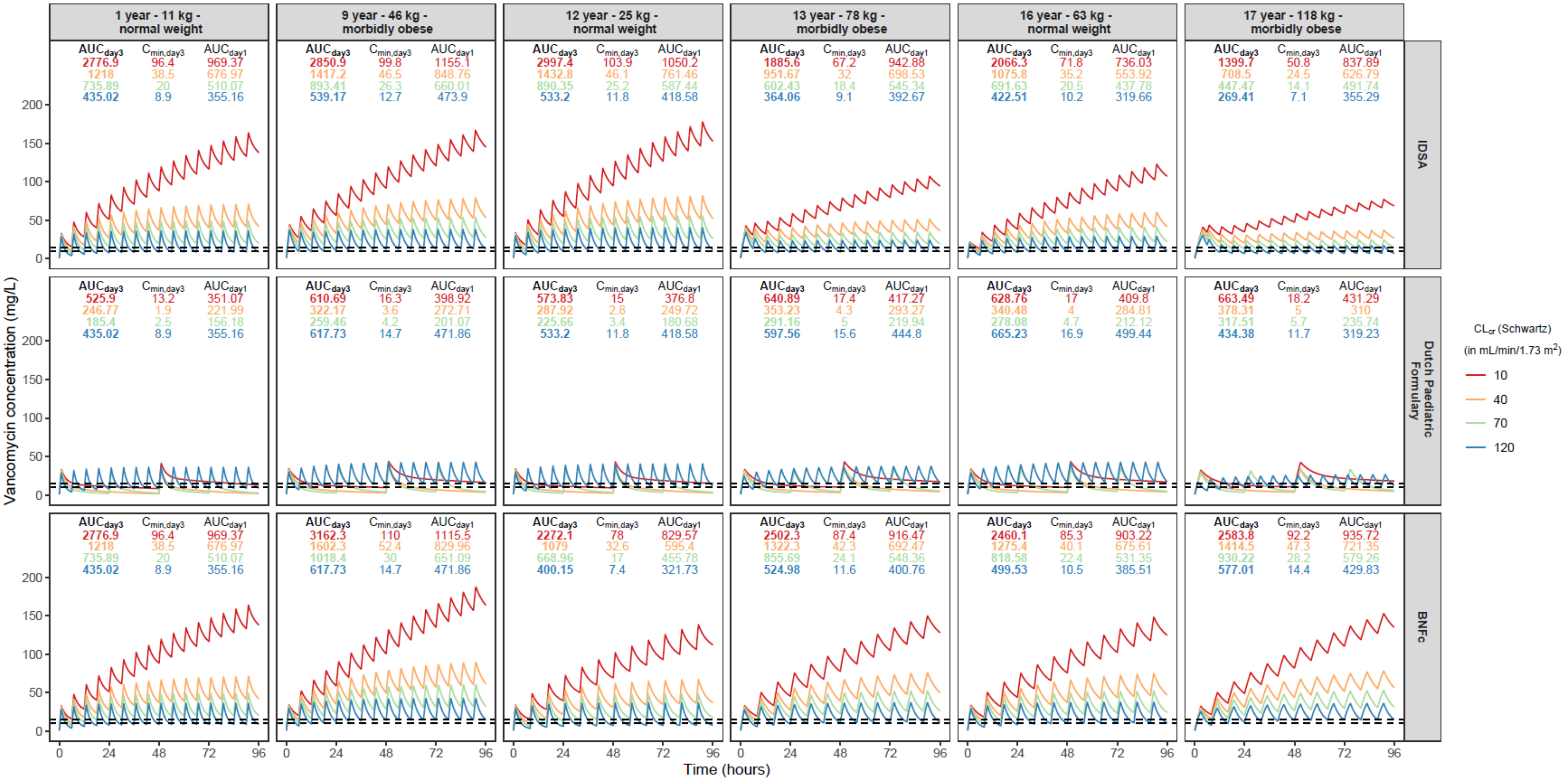

Figure S3. Vancomycin concentrations (mg/L) versus time (hours) in several typical individuals with bodyweight ranging 10 – 120 kg and renal function ranging 10 – 120 ml/min/1.73 m^2 where vancomycin is dosed according to three existing dosing guidelines: Infectious Diseases Society of America (IDSA, upper panel), Dutch Paediatric Formulary (middle panel) and British National Formulary for Children (BNFc, lower panel). For each individual, AUC (in bold), $C_{min,}$ at day 3, as well as AUC at day 1 is shown in the graph (where colour correspondents to the individual's renal function). Dashed lines represent the target concentrations for the trough concentrations (10 – 15 mg/L). *AUC* area under the curve, C_{min} minimum (trough) concentration.

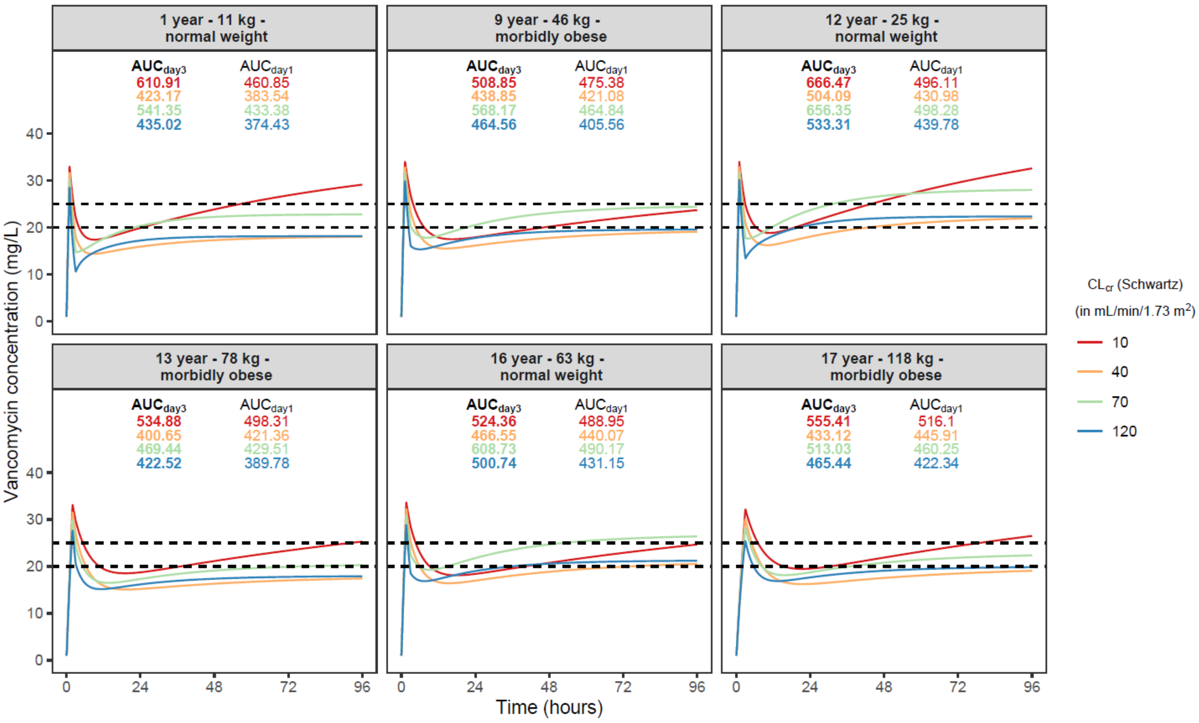

Figure S4. Vancomycin concentrations (mg/L) versus time (hours) in different typical individuals with body weight ranging 10 – 120 kg and renal function ranging 10 – 120 ml/min/1.73 m^2 where vancomycin is dosed as continuous infusion according to the proposed dose nomogram (Table 3). Here, the first dose of 15 mg/kg was given as a loading dose, after 3 hours followed by the proposed daily maintenance dose given as a 24 h infusion. For each individual, AUC at day 3 (in bold), as well as AUC at day 1 is shown in the graph (where colour corresponds to the individual's renal function). Dashed lines represent the target concentrations for continuous infusion (20 – 25 mg/L). *AUC* area under the curve.

6. NONMEM control stream for the final model

```
$PROBLEM VANCO 1-18

$INPUT ID TIME AMT RATE DV=DROP LNDV=DV MDV LLOQ BLQ OCC
    EVID HT WT LBW_AS LBW_P BSA WTAGE WTEXS BMI GRP
    AGED AGEG SEX RACE RRT ICU HOSP TIM0 TAD CREAT CREAT_TV
    CREAT_FIRST CREAT_BL SCHW SCHW_FIRST SCHW_di SCHW_di_FIRST
    SCHW_BL SCHW_BL_di SCHW_SL SCHW_di_SL CREAT_IMP NEUT NPEN
    RIFLE NEPHROTOX NPENE CRP LYMPH SCHW_GRP

$DATA nonmem_1_18J_NOCB_SCHW_GRP.prn IGNORE=# IGNORE=(RRT.EQ.1) IGNORE=(BLQ.EQ.1)
IGNORE=(WT.LT.0)

$SUBROUTINE ADVAN3 TRANS4

$PK

SCHW_MAX=SCHW

IF(SCHW.GT.120) SCHW_MAX=120

CREAT_RATIO=CREAT/CREAT_TV

;

IF (WT.GT.0) THEN

        TVCL_WT     = THETA(1) * ((WT/22.1)**THETA(6)); TVCL_WT

        TVV1        = THETA(2)*((WT/22.1)**THETA(7))

        TVQ         = THETA(3)*((WT/22.1)**THETA(8))

        TVV2        = THETA(4)*((WT/22.1)**THETA(7))

    ELSE

        TVCL_WT     = THETA(1)

        TVV1        = THETA(2)

        TVQ         = THETA(3)

        TVV2        = THETA(4)

    ENDIF

;

TVCL = TVCL_WT*((SCHW_MAX/100)**THETA(5))

;
```

```
CL = TVCL*EXP(ETA(1))

V2 = TVV2*EXP(ETA(2))

;

V1 = TVV1*EXP(ETA(3))

Q = TVQ*EXP(ETA(4))

 ;

S1   = V1 ;

;

ET1=ETA(1)

ET2=ETA(2)

ET3=ETA(3)

ET4=ETA(4)


$THETA

(0, 2.12) ; TVCL_WT

(0, 8.87) ; TVV

(0, 1.54) ; Q

(0, 12) ; V2

(1) FIX ; CL_SCHW EXP

(0.753) ; CL_WT_EXP

(1) FIX ; EXP V1_V2_WT

(0,0.75) ; Q_WT_EXP


$OMEGA BLOCK(2)

 0.0822  ; CL ETA 1

 -0.0313 ; COVAR ET1-ET2,

 0.67  ; V2 ETA 2
```

```
$OMEGA
 0 FIX  ;V1 ETA 3
 0 FIX  ;Q ETA 4

$ERROR

IPRED=0
IF(F.GT.0) IPRED  = LOG(F)

IRES   = DV - IPRED
W      = F
IF(W.EQ.0) W = 1
IWRES = IRES/W

Y     = IPRED+ERR(1) ;

$SIGMA
 0.0788 ; PROP ERR IN LOGDOMAIN

$ESTIMATION METHOD=1 INTER MAXEVAL=9999 POSTHOC ;
$COVARIANCE PRINT=E ;
$TABLE ESAMPLE=10000 ID TIME IPRED IWRES CWRES AMT TVCL CL TVV1 V1 TVQ Q TVV2 V2
ET1 ET2 ET3 ET4 NPDE MDV BLQ LLOQ CREAT_RATIO HT WT LBW_AS LBW_P BSA WTAGE WTEXS
BMI GRP AGED AGEG SEX RACE RRT ICU HOSP TIM0 TAD CREAT CREAT_FIRST CREAT_BL SCHW
SCHW_GRP SCHW_FIRST SCHW_di SCHW_di_FIRST SCHW_BL SCHW_BL_di SCHW_SL SCHW_di_SL
CREAT_IMP NEUT NPEN RIFLE NEPHROTOX NPENE CRP LYMPH NOPRINT ONEHEADER
```

7. References used in the supplementary file

1. Van Rongen A, Van der Aa MP, Matic M, et al.: Increased Metformin Clearance in Overweight and Obese Adolescents: A Pharmacokinetic Substudy of a Randomized Controlled Trial. *Paediatr Drugs* 2018; 20:365–374

2. Centers for Disease Control and Prevention (CDC): Clinical Growth Charts [Internet]. [cited 2017 Sep 25] Available from: https://www.cdc.gov/growthcharts/clinical_charts.htm

3. World Health Organisation: WHO Child Growth Standards [Internet]. [cited 2017 Sep 25] Available from: https://www.who.int/childgrowth/standards/en/

4. Mosteller RD: Simplified calculation of body-surface area. *N Engl J Med* 1987; 317:1098

5. Al-Sallami HS, Goulding A, Grant A, et al.: Prediction of Fat-Free Mass in Children. *Clin Pharmacokinet* 2015; 54:1169–78

6. Peters AM, Snelling HLR, Glass DM, et al.: Estimation of lean body mass in children. *Br J Anaesth* 2011; 106:719–23

7. Ceriotti F, Boyd JC, Klein G, et al.: Reference intervals for serum creatinine concentrations: assessment of available data for global application. *Clin Chem* 2008; 54:559–66

8. Schwartz GJ, Work DF: Measurement and estimation of GFR in children and adolescents. *Clin J Am Soc Nephrol* 2009; 4:1832–43

9. Beal SL, Sheiner LB, Boeckmann A: NONMEM user's guide. University of California, San Francisco, California. 1999.

10. Keizer RJ, Karlsson MO, Hooker A: Modeling and Simulation Workbench for NONMEM: Tutorial on Pirana, PsN, and Xpose. *CPT pharmacometrics Syst Pharmacol* 2013; 2:1–9

11. Lindbom L, Pihlgren P, Jonsson EN, et al.: PsN-Toolkit--a collection of computer intensive statistical methods for non-linear mixed effect modeling using NONMEM. *Comput Methods Programs Biomed* 2005; 79:241–57

12. Wang C, Peeters MYM, Allegaert K, et al.: A bodyweight-dependent allometric exponent for scaling clearance across the human life-span. *Pharm Res* 2012; 29:1570–1581

13. Bartelink IH, Boelens JJ, Bredius RGM, et al.: Body weight-dependent pharmacokinetics of busulfan in paediatric haematopoietic stem cell transplantation patients: towards individualized dosing. *Clin Pharmacokinet* 2012; 51:331–45

14. De Cock RFW, Allegaert K, Brussee JM, et al.: Simultaneous pharmacokinetic modeling of gentamicin, tobramycin and vancomycin clearance from neonates to adults: Towards a semi-physiological function for maturation in glomerular filtration. *Pharm Res* 2014; 31:2643–2654

15. Van Rongen A, Vaughns JD, Moorthy GS, et al.: Population pharmacokinetics of midazolam and its

metabolites in overweight and obese adolescents. *Br J Clin Pharmacol* 2015; 80:1185–1196

16. Bergstrand M, Hooker AC, Wallin JE, et al.: Prediction-corrected visual predictive checks for diagnosing nonlinear mixed-effects models. *AAPS J* 2011; 13:143–51

17. Dosne A-G, Bergstrand M, Harling K, et al.: Improving the estimation of parameter uncertainty distributions in nonlinear mixed effects models using sampling importance resampling. *J Pharmacokinet Pharmacodyn* 2016; 43:583–596

18. Rybak MJ, Le J, Lodise TP, et al.: Therapeutic monitoring of vancomycin for serious methicillin-resistant Staphylococcus aureus infections: A revised consensus guideline and review by the American Society of Health-System Pharmacists, the Infectious Diseases Society of America, the Pediatr. *Am J Health Syst Pharm* 2020; 77:835–864

19. Nederlands Kenniscentrum voor Farmacotherapie bij Kinderen: Dutch Paediatric Formulary/Kinderformularium [Internet]. [cited 2020 Mar 15] Available from: https://www.kinderformularium.nl/

20. Paediatric Formulary Committee: British National Formulary for Children (BNFc) (online) [Internet]. [cited 2020 Mar 20] Available from: http://www.medicinescomplete.com
